# What applications for plasma endocan measurement in intensive care? A clarification

**DOI:** 10.1186/s13054-023-04686-1

**Published:** 2023-10-17

**Authors:** Victoria Dubar, Camille Chenevier-Gobeaux, Julien Poissy, Alexandre Gaudet

**Affiliations:** 1 Pôle de Médecine Intensive – Réanimation, Hôpital Roger Salengro, CHU Lille, 59000 Lille, France; 2 Department of Biochemistry / Automated Biological Diagnostic, Cochin Hospital, APHP-Centre Université de Paris, CEDEX 14, 75679 Paris, France; 3 Inserm U1285, CHU Lille, CNRS, UMR 8576, UGSF, Unité de Glycobiologie Structurale Et Fonctionnelle, Univ. Lille, 59000 Lille, France; 4 U1019 – UMR 9017 – CIIL – Center for Infection and Immunity of Lille, Univ. Lille, 59000 Lille, France

To the editor,

The publications concerning the measurement of plasma endocan in critically ill patients have gradually generated hypotheses regarding its potential clinical utility. However, it may be useful to revisit the main applications that have been proposed to date and their applicability in daily practice (Fig. 1).

**Fig. 1:**
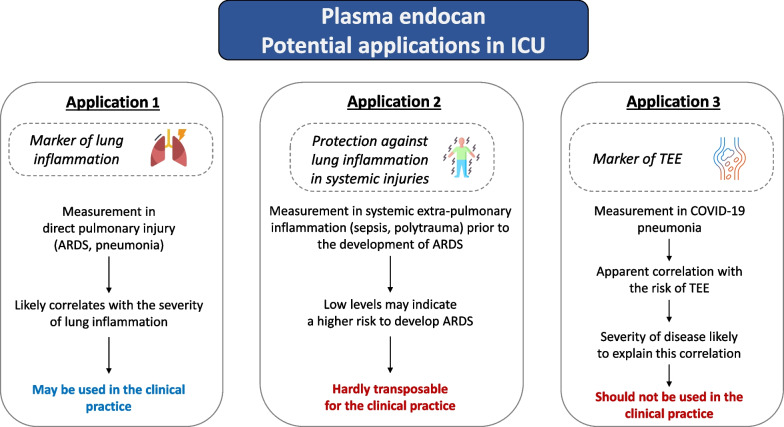
Plasma endocan: potential applications in ICU. *ARDS* Acute respiratory distress syndrome, *TEE* Thrombo-embolic events

Endocan is an endothelial-cell secreted proteoglycan with notable expression in the lung in human, upregulated under the influence of pro-inflammatory cytokines, such as TNF-α or IL-1β. Besides, consistent data suggest an immunomodulator effect of endocan, supposedly explained by its ability to regulate the recruitment of immune cells to the site of inflammation, especially in the lung [1].

The first utility that can be identified is that of a sensor for pulmonary inflammation. This has been described several times in the context of established acute respiratory distress syndrome (ARDS) [2–5], as well as community-acquired [6] or healthcare-associated pneumonia [7]. The clinical interest highlighted by the authors lies notably in the prognostic nature of the biomarker, aiming to detect early signs of deterioration in certain patients.

During the recent COVID-19 pandemic period, some studies have shown that endocan could be valuable in early detection of high-risk patients for acute respiratory failure requiring intubation [8]. Other authors have also proposed the hypothesis that measuring endocan in the post-operative context of cardiac surgeries could help detect the onset of subclinical pneumonias [7]. This approach of considering endocan merely as a marker of pulmonary inflammation seems to be the one that gathers the most supporting evidence to date.

A second application that has been studied is that of “anti-inflammatory endocan”. Indeed, experimental data suggest that endocan, an anti-inflammatory molecule secreted notably in the lung, may play a protective role against potential respiratory complications in extra-pulmonary systemic inflammation. In this context, insufficiently high levels of endocan, measured before the onset of pulmonary inflammation in a situation of systemic aggression, could thus reflect a defect in the modulation of inflammation in the lung, resulting in a high risk of ARDS [9–11]. However, this potential application, even if the underlying hypothesis were confirmed, appears to have limited applicability in routine practice. Indeed, the heterogeneity of systemic insults like sepsis, both in nature and intensity, complicates the task of distinguishing low endocan levels that correspond to the identification of truly hyper-inflammatory patients with insufficient endocan elevation from those who simply have a low-intensity systemic inflammation [12].

The third potential application has been highlighted in a previous correspondence by Honoré et al*.,* who questioned the mechanisms underlying the apparent correlation between high blood levels of endocan and severity in COVID-19 related acute respiratory distress syndrome (ARDS) [13]. On this occasion, the authors proposed that endocan could be a witness of genuine lung inflammation, as opposed to its utility as a potential marker of thrombo-embolic events (TEE), particularly in the context of COVID-19.

This latter hypothesis was based on previously published results, including one study by Chenevier-Gobeaux et al*.* conducted in the setting of COVID-19, reporting the association between thrombo-embolic events and high blood levels of endocan [14, 15]. However, it should be noted that this cohort included a wide range of profiles regarding COVID-19 severity, with a majority of patients who did not require admission to the intensive care unit (ICU). Conversely, in another study published by Dubar et al., endocan blood levels measured on ICU admission in critically ill COVID-19 patients were not different between patients with TEE and those with no TEE [16]. In contrast, D-Dimers were actually found more elevated in case of TEE than in subjects with no TEE. Previous results reporting a statistical association between higher blood levels of endocan and more frequent TEE in the setting of COVID-19 are therefore likely to reflect a greater severity of ARDS. Interestingly, when pooling the data from Dubar et al. (100 patients with no TEE and 24 patients with TEE) [16] with those from critically ill subjects included in the study published by Chenevier-Gobeaux et al*.* (13 patients with no TEE and 11 patients with TEE) [14], we find that blood levels of endocan in critically ill COVID-19 patients with TEE (median [IQR] 5.8 [4.1; 10.9] ng/mL) do not differ from those found in patients with no TEE (median [IQR] 7.6 [5; 14.8] ng/mL) (*p* = 0.11 by Mann–Whitney test). In this respect, we feel that endocan should remain an inflammatory marker in the strict sense of the term, helpful to categorize the ARDS phenotype, rather than a marker of TEE, particularly in the setting of severe COVID-19.

In total, the use of endocan as a prognostic and phenotypic marker for established acute pulmonary inflammation appears to be the application with the most scientifically supported foundation and the highest potential for clinical practice applicability. However, this application needs further validation as no clinical trial has assessed to date the benefit of measuring plasma endocan for critically ill subjects. Besides, the possibility that plasma endocan measurement could lead to harmful decisions through unknown pathways has never been ruled out and therefore needs to be evaluated. Further, other potential applications also seem of interest and deserve exploration in the future. Monitoring endocan as a marker for treatment response in ARDS or pneumonia, to our knowledge, has not been evaluated to date and could represent an interesting avenue of research for the future.

## Data Availability

Not applicable.

## Abbreviations

ARDS: Acute respiratory distress syndrome
ICU: Intensive care unit
TEE: Thrombo-embolic event
